# Unassigned complex unique recombinant forms related to CRF36_cpx in children identified in an HIV-1 outbreak in Pakistan

**DOI:** 10.1089/AID.2021.0168

**Published:** 2022-07-26

**Authors:** Syed Hani Abidi, Dilsha Siddiqui, Syed Faisal Mahmood, Rehana Siddiqui, Apsara Ali Nathwani, Aneeta Hotwani, Sharaf Ali Shah, Palwasha Khan, Rashida Abbas Ferrand, Fatima Mir

**Affiliations:** 1 Department of Biological and Biomedical Sciences, Aga Khan University, Karachi, Pakistan; 2 Department of Biomedical Sciences, Nazarbayev University School of Medicine, Nur-Sultan, Kazakhstan; 3 Department of Medicine, Aga Khan University, Karachi, Pakistan; 4 Department of Community Health Sciences, Aga Khan University, Karachi, Pakistan; 5 Department of Pediatrics and Child Health, Aga Khan University, Karachi, Pakistan; 6 Bridge Consultants Foundation, Karachi, Pakistan; 7 Department of Clinical Research, London School of Hygiene & Tropical Medicine, London, UK

**Keywords:** HIV-1, Unique Recombinant Form, Drug Resistance, Pakistan, children

## Abstract

In 2019, an outbreak of HIV infection predominantly affecting children occurred in Larkana district, Pakistan. This is the largest outbreak ever reported in this age group in Pakistan. In this study, we report two HIV-1 unique recombinant forms identified within the outbreak. Blood samples were collected from HIV-positive children as part of a case-control study to investigate the outbreak. Pol gene was sequenced and used to detect HIV subtype/recombinant forms using subtype, recombination, and phylogenetic analyses. Drug resistance mutation (DRM) analysis was performed to characterize the drug DRMs in each sequence. We observed the emergence of two unassigned unique recombinant forms related to CRF36_cpx in 15 individuals out of 344 samples. Genotype analysis revealed the presence of multiple DRMs associated with resistance to reverse transcriptase inhibitors. The discovery of these unassigned URFs in our population highlights the need for comprehensive molecular epidemiological studies to fully understand the distribution and drug resistance patterns to aid control efforts.

## Introduction

HIV-1 is a remarkably diverse retrovirus, with the Group M of HIV-1 classified into nine subtypes A-D, F-H, J, and K.^1^ HIV-1 is geographically diverse with reference to its subtypes.^1^ For instance, in America, Australia, and Europe subtype B is predominant; across East Africa subtype A infection is dominant; in West Africa, almost all other known subtypes are known to be circulating and CRF02_AG and subtype G are the predominant subtypes. Similarly, Subtype C is the predominant subtype in Southern Africa, Ethiopia, and India.^2^ Subtype A1 is predominant in Pakistan followed by subtype B, CRF02_AG, and A1G.^3^

HIV infection with multiple strains can occur in an individual with repeated exposure e.g. due to high-risk sexual behavior and can result in the emergence of new or complex recombinant forms.^4^ Over 100 circulatory and unique recombinant forms (CRFs and URFs) of HIV-1 have been identified. Approximately 20% of HIV-1 infections worldwide are caused by URFs, while in some African countries this proportion is as high as 40%.^5^

In Pakistan, HIV-1 currently exists as a concentrated epidemic in certain high-risk groups, such as persons who inject drugs (PWID) and men who have sex with men (MSM), and transgender sex workers (*Hijra* sex workers), with frequent spillovers into low-risk populations in form of isolated outbreaks.^6–9^ Molecular epidemiological studies from Pakistan have shown the presence of diverse subtypes and URFs in the country, namely B, C, D, G, A1D, A1G, 01G, CG, 01_AE, CRF02A1, CRF02_AG, and CRF35_AD, while subtype A1 is the predominant subtype.^3, 10^ In addition to these known subtypes, several new and unique CRFs and URFs have been reported: URF_DG^11^, CRF56_cpx, and CRF02_A1.^8, 12^

In April 2019, an outbreak of HIV-1 infection predominantly affecting children was reported in Larkana district, Sindh province, Pakistan. This is the largest outbreak ever reported in this age group in Pakistan, where more than 1000 children had tested positive for HIV-1.^13^ As part of the investigation of the Larkana outbreak, epidemiological and molecular studies were carried out. ^14^ In this study, we report two unassigned URFs related to CRF36_cpx from individuals identified with HIV infection in the outbreak that possibly emerged from recombination between CRF02_AG and CRF36_cpx and CRF02_AG, CRF36_cpx and subtype H, respectively.

## Methods

As part of a case-control study to investigate the outbreak, blood samples were collected from children aged below 15 years diagnosed with HIV-1 infection (cases) in Larkana, and uninfected age and sex-matched community controls.^15^ The samples were collected after obtaining informed consent from the participants and/or from the guardians of the participant.^16^ A questionnaire was used to obtain demographics and relevant risk factor information from the study participants. The study was approved by the Aga Khan University Ethical Review Committee (ERC# 2019-1536-4200), and all experiments were performed in accordance with relevant guidelines and regulations. In this study, we focus on two unassigned complex URFs. The findings of the complete molecular epidemiological analysis will be reported separately.

Following DNA extraction from 344 blood samples collected from cases, the *pol* (covering protease and reverse transcriptase region) gene of HIV-1 was amplified using two sets of primer using a two-step nested polymerase chain reaction (PCR). Two sets of outer primers were used, forward (POLOF CAGCATGYCAGGGAGTRGGRGGACC, nt; 1832-1856, HXB2, IBF1 5’-AAATGATGACAGCATGTCAGGGAGT-3’. nt 1823-1847, HXB2) and reverse (IBR1 5’-AACTTCTGTATATCATTGACAGTCCA-3’. nt 3303-3328, HXB2); the first-round product was used as a template in the second round with primer set, Forward (POLIF 5’-AGGCTAATTTTTTAGGGAARATYTGGCCTTCC-3’. nt 2078-2109, HXB2) and Reverse (RTOUT3 5’-TATGTCATTGACAGTCCAGCT-3’. nt 3300-3320, HXB2). PCR Mastermix (ABM) Bestaq (2X) and Hotstart (2X) were used to prepare a 25μl reaction mixture with primer 0.8 pmol and 0.6 pmol for the first and second round, respectively. Thermocycling conditions were as follows: 95°C for 5 min was followed by 40 cycles of denaturation at 95°C for 1 min, annealing at 50°C for IBF1/IBR1 and 55°C for POLOF/IBR1 sets (round 1), 60°C (round 2) for 20 seconds, and extension at 72°C for 1 min with a final extension of at 72°C for 7 min. For confirmation/validation, each run included positive and negative controls. The amplicons were subsequently sequenced on a Sanger sequencing platform (Macrogen, Korea).

The sequences (n=15) were submitted to Genbank and were assigned the accession numbers MN698251, MN698252, MN698253, MN698255, MN698256, MN698257, MN698258, MN698259, MN698260, MN698261, MN698262, MN698263, MN698264, MN752136, MN752137.

The obtained *pol* sequences were subtyped using the HIV-1 REGA subtyping tool, while the jpHMM tool was used to detect recombinations and recombination breakpoints within the pol gene of the HIV-1 genome. ^12, 13, 15, 17^ The strain identity was investigated using phylogenetic analysis. For this, a multiple sequence alignment (MSA), containing outbreak sequences (ID: AKULO) as well as HIV-1 subtype reference obtained from the Los Alamos HIV sequence Database, was generated using the MAFFT program.^18, 19^ For 1 sequence (AKULO_387), for which two recombination breakpoints were predicted by the jpHMM, two separate MSAs were generated. The first and second MSA of the AKULO_387 sequence comprised split nucleotides spanning positions 2236-3192 and 3193-3313 (with reference to the HXB2 genome) respectively. These two MSAs were aligned separately with the HIV-1 subtype reference obtained from the Los Alamos HIV sequence Database using the MAFFT online MSA tool. Subsequently, all three MSAs were used to generate individual Maximum Likelihood (ML) phylogenetic trees using PhyML software 3.0 (http://www.atgc-montpellier.fr/phyml/) with a Generalized Time Reversible (GTR) model of nucleotide substitution and approximate likelihood ratio test (aLRT) and the Shimodaira–Hasegawa (SH)-aLRT measure of branch support. Finally, genotyping and drug resistance analysis of the AKULO sequences was performed using the Stanford drug resistance database (HiVdb Program).^15^

## Results

**Fig. 1:**
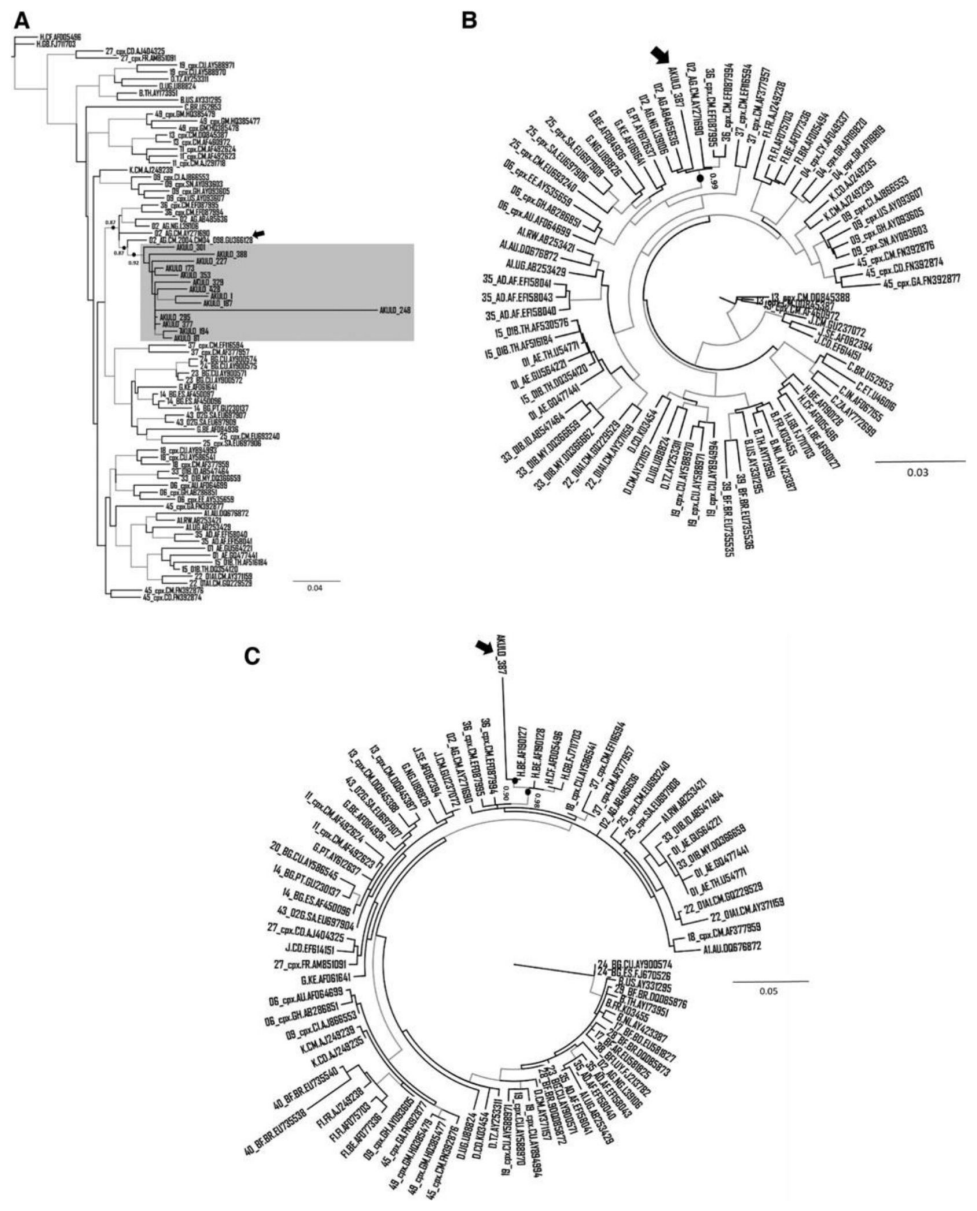
Phylogenetic analysis of unassigned URFs (A) ML phylogenetic tree for 14 outbreak sequences represented by the gray box and the Cameroonian CRF02_36cpx recombinant sequence indicated by the black arrow. ML phylogenetic tree for 1 outbreak sequence (AKULO_387) constructed using MSAs (developed on the breakpoints predicted by jpHMM) spanning nucleotides (B) 2236–3192 and (C) 3193–3313 (with reference to the HXB2 position). The AKULO_387 sequence is shown by the black arrow. The node support values of the clusters of interest (A–C). The gray-colored branches represent the aLRT SH-like support values ‡0.80. aLRT, approximate likelihood ratio test; ML, maximum likelihood; SH, Shimodaira–Hasegawa; URF, unique recombinant form.

Of the 344 samples from cases, unassigned URFs were observed in 15. The median age of the 15 participants was 2.8 years (range: 0.8-9 years) and 33% were female. At the time of sample collection, two participants were ART-naïve, and the remainder had only recently started ART (Table 1). Subtyping analysis identified the 15 sequences as CRF02_AG with undefined recombination related to CRF36_cpx. On analysis, 14 sequences clustered with CRF36_cpx sequences submitted from Cameroon (accession numbers: GU366128, EF087994, and EF087995) and CRF02_AG sequences submitted from Liberia and Nigeria (accession numbers: AB485636, L39106, respectively) (Figure 1A). These 14 sequences exhibited a strong node value of 0.87 (Figure 1A), indicating these sequences to be an unassigned recombinant form related to CRF36_cpx, possibly emerging from recombination between CRF02_AG and CRF36_cpx. The Cameroonian URF (accession number: GU366128) most closely clustering with the 14 sequences generated in our study (Figure 1A, Black arrow) was previously reported from a 2004 cohort study where the authors identified this strain as CRF02_AG containing 36cpx recombination at the 5’end of the pol region.^20^ Interestingly, when we included this sequence in our phylogenetic tree, it became part of the larger cluster (node support value 0.87) that contained outbreak sequences as well as CRF02_AG and CRF36_cpx sequences (Figure 1A), further supporting that the 14 outbreak sequences to be unassigned URF related to CRF36_cpx.

For the remaining one sequence (ID: AKULO_387) the phylogenetic analysis was performed using the two MSAs developed on the breakpoints predicted by jpHMM. On the phylogenetic tree, based on the first MSA, this sequence clustered with CRF02_AG submitted from Cameroon, Nigeria, and Liberia (accession number: AY271690, L39106, AB485636, respectively) and CRF36_cpx submitted from Cameroon (accession number: EF087994-EF087995) with node support value of 0.99 (Figure 1B). Similarly, on the phylogenetic tree, based on the second MSA the sequence clustered with subtype H, submitted from Belgium (accession number: AF190127) with node support value of 0.90 (Figure 1C), indicating the strain to be a subtype H-like unassigned complex URF related to CRF36_cpx.

**Figure 2:**
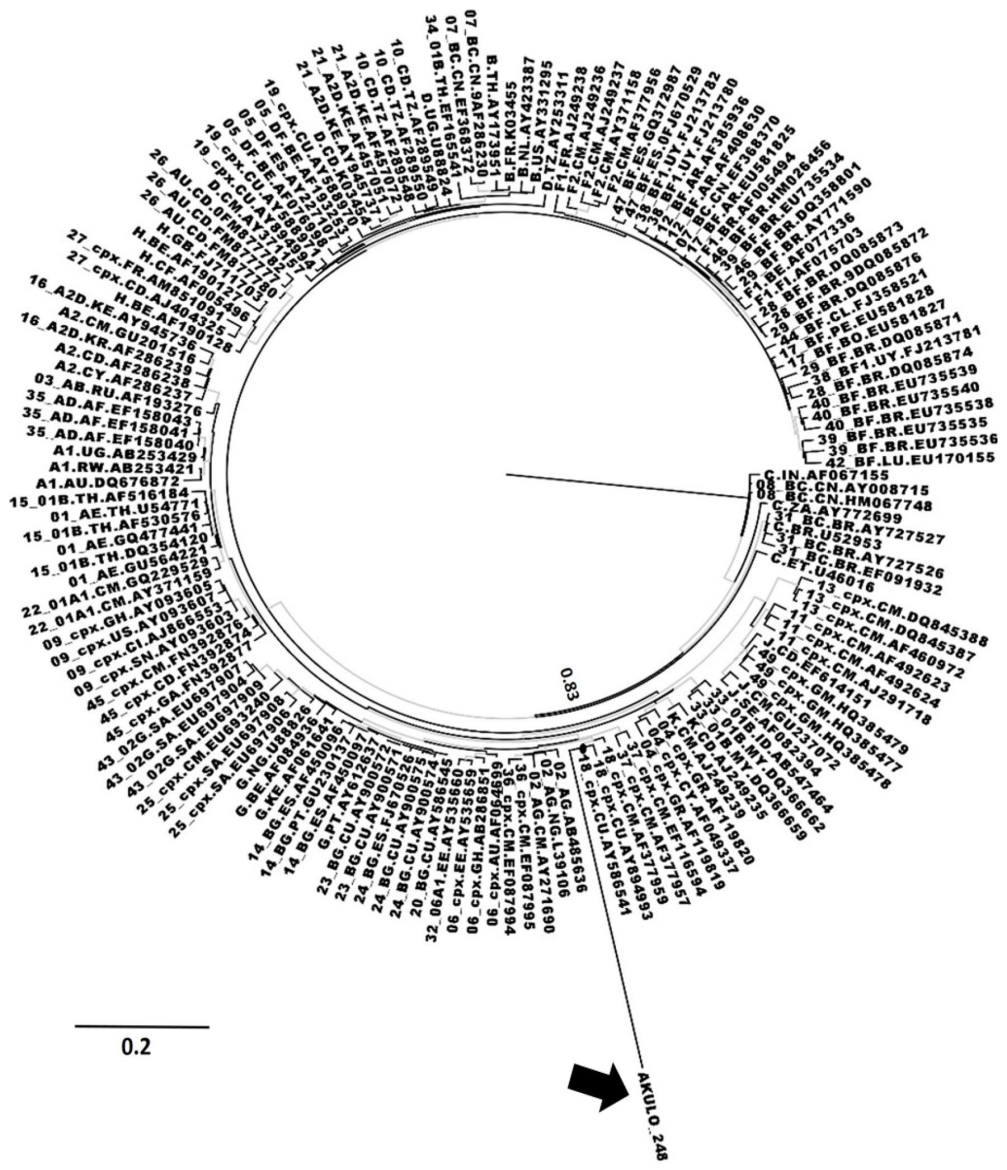
Phylogenetic analysis of unassigned URF in AKULO 248 Phylogenetic analysis of unassigned URF in AKULO 248: ML phylogenetic tree showing the AKULO_248 sequence (red color). The blue-colored branches represent the aLRT SH-like support values ‡0.80. The AKULO_248 sequence is shown by the black arrow.

The branch length of the sequence in one of the 14 samples (ID: AKULO_248) in the cluster (Figure 1A) suggested the presence of additional recombination(s) that was not predicted by any tool. The phylogenetic analysis of this sequence showed clustering with CRF18_cpx in addition to CRF02_AG and CRF36_cpx (Figure 2), suggesting additional recombination; however, this warrants further investigation.

On genotypic analysis, six of the fifteen (40%) sequences contained mutations associated with resistance to antiretroviral drugs (Table 1): the T215N, M184V, and Y115F mutations are associated with resistance to nucleoside reverse transcriptase inhibitors (NRTIs); the K103N, Y181C, A98G, V179L mutations are associated with resistance to non-nucleoside reverse transcriptase inhibitors (NNRTIs). No protease inhibitor resistance mutations were observed.^19^ A similar analysis of the reference sequences that clustered with our outbreak sequences (Figure 1) showed that none of the reference sequences harbored any DRM, except subtype H sequence (accession no: AF190127, Figure 1C) that had DRMs D67N, K70R, K219Q associated with resistance to all NRTIs.

## Discussion

The current study reports the detection of novel unassigned URFs related to CRF36_cpx in children identified with HIV-1 during the Larkana outbreak. CRF 02_AG is one of the main circulating strains in Pakistan; however, the current analysis suggests that it is recombining with other subtypes/CRFs, leading to the emergence of subtype H-like or CRF36_cpx related URFs. CRF36_cpx is limited to Cameroon and has been found to recombine with CRF02_AG.^20^ Subtype H has only been reported in Central Africa and the United Kingdom.^19, 21, 22^ Recombination of subtype H with multiple other subtypes and CRFs has been observed including recombination with CRF04_cpx, CRF27_cpx, and complex recombination with U/CRF02_AG.^19, 23^ One limitation of this study is the use of pol sequence only for the assignment of the recombinant form. Analysis based on longer regions of the HIV-1 genome or whole genome, which could not have been done due to funding constraints, would have been useful to accurately determine the recombinant form and the region(s) of recombination. Nonetheless, our analysis supports the presence of subtype-H like or CRF36_cpx related URFs, which suggest that the HIV epidemic in Pakistan is much more dynamic and complex than previously thought. Some of these strains may become more adaptive and emerge as major forms in the future.

Genotypic analysis of sequences suggested the presence of drug resistance mutations that cause resistance against multiple reverse transcriptase inhibitors. Three out of 13 ARV-experienced individuals harbored mutations associated with high-level resistance to NRTI and/or NNRTIs DRMs (M184V, K103N, Y115F, and Y181C). Two of the 13 individuals also had mutations T215N and Y179L associated with resistance against zidovudine, and abacavir and tenofovir, respectively, which are the first-line regimen in Pakistan. The mutation T215N is a revertant mutation; the presence of this mutation suggests that the individual was previously infected with or had acquired HIV-1 whose majority population had T215Y/F (a highly resistant NRTI mutation).^9^ Similarly, the mutation A98G (observed in 2 individuals) is associated with resistance to multiple NNRTIs. No drug resistance mutations were observed in ARV-naïve individuals. While all but two individuals were ART-experienced, the remainder had started ART recently (mean ARV duration was 1 month; range 8 days to 3 months). Therefore, it is unlikely that these mutations would have emerged as a result of sub-optimal adherence and may be due to transmitted drug resistance.^24, 25^ This may have possible implications for the treatment and control of HIV in Pakistan.

The presence of multiple drug resistance mutations in these strains, especially to first-line ART drugs, is alarming as it limits treatment options. Large-scale transmission of resistant strains can hamper efforts to control the spread of the HIV epidemic in Pakistan, where second-line drugs are not easily available. The discovery of multiple URFs/ CRFs in this outbreak highlights the need for comprehensive molecular epidemiological studies and molecular surveillance to understand the distribution of different genotypes as well as origin, transmission, and drug resistance patterns. This will inform the appropriate treatment of individuals with HIV and strategies for preventing further outbreaks and controlling the spread of the HIV epidemic in the country.

## Figures and Tables

**Table 1:** Data and drug resistance mutation (DRM) profile of 15 individuals ART drugs affected by mutations are shown in superscript. **Abbreviations:** LNZ: Lamviduine, Nevirapine, Zidovudine. Nucleoside Reverse Transcriptase Inhibitor (NRTI): Emtricitabine (FTC), Lamivudine (3TC), Abacavir (ABC), Tenofovir disproxil fumarate (TDF), Zidovudine (AZT). Non-Nucleoside Reverse Transcriptase Inhibitor (NNRTI): Efavirenz (efv), Nevirapine (nvp), Etravirine^26^, Rilpivirine (rpv), Doravirine (dor). **Key: Bold font =** High level resistance; *=intermediate level resistance; ^=low level resistance; ♢= potential low-level resistance. Different drug resistance levels are separated by / sign. ‘-’ sign indicates the absence of any DRM.

Sample ID	Gender	Age (Years)	ART	Therapy Duration	Drug Resistance Mutations		
					PI	NRTI	NNRTI
**AKULO_1**	Male	1.6	LNZ	11 days	-	-	-
**AKULO_81**	Female	0.8	LNZ	20 days	-	-	-
**AKULO_173**	Male	1.9	LNZ	1 month	-	-	-
**AKULO_187**	Male	4	LNZ	1.25 months	-	-	-
**AKULO_194**	Female	1.4	LNZ	1 month	-	-	K103N^**efv,nvp**^, V179L^rpv^/efv,etr,nvp♢^
**AKULO_227**	Female	3	LNZ	8 days	-	Y115F^**abc**/tdf^^	-
**AKULO_248**	Male	9	Naïve	N/A	-	-	-
**AKULO_295**	Male	3.2	LNZ	1.75 months	-	-	-
**AKULO_301**	Female	7.3	LNZ	2 months	-	-	-
**AKULO_329**	Female	3	Naïve	N/A	-	-	-
**AKULO_353**	Male	3.1	LNZ	1.5 months	-	-	A98G^nvp*/dor,efv,rpv^/etr♢^
**AKULO_377**	Male	2.6	LNZ	1 month	-	M184V^**ftc,3tc**/abc^^	K103N^**efv,nvp**^ Y181C^**nvp**/efv,etr,rpv*^
**AKULO_387**	Male	1.3	LNZ	2.5 months	-	T215N ^azt^^	-
**AKULO_388**	Male	1	LNZ	3 months	-	-	A98G^nvp*/dor,efv,rpv^/etr♢^
**AKULO_428**	Male	3	LNZ	3 months	-	-	-
